# Should patents for antiretrovirals be waived in the developing world? Annual varsity medical debate - London, 21 January 2011

**DOI:** 10.1186/1747-5341-6-13

**Published:** 2011-07-08

**Authors:** Fenella Corrick, Robert Watson, Sanjay Budhdeo

**Affiliations:** 1 Oriel College, University of Oxford, Oriel Square, Oxford, OX1 4EW, UK; 2 Green Templeton College, University of Oxford, Woodstock Road, Oxford, OX2 6HG, UK; 3 New College, University of Oxford, Holywell Street, Oxford, OX1 3BN, UK

## Abstract

The 2011 Varsity Medical Debate, between Oxford and Cambridge Universities, brought students and faculty together to discuss the waiving of patents for antiretroviral therapies in the developing world. With an estimated 29.5 million infected by Human Immunodeficiency Virus (HIV) in low- and middle-income countries and only 5.3 million of those being treated, the effective and equitable distribution of anti-retroviral therapy (ART) is an issue of great importance. The debate centred around three areas of contention. Firstly, there was disagreement about whether patents were the real barrier to the access of anti-retroviral therapy in the developing world. Secondly, there were differing views on the effectiveness of a patent pool. Thirdly, concerns were raised over the impact of waiving patents on research to produce new and better anti retro-viral drugs.

## Background

After the success of the 2010 Varsity Medical Debate [1], the Third Varsity Medical Debate between Cambridge and Oxford Universities took place on Friday 21^st ^January 2011, hosted by the Royal College of General Practitioners. A prestigious panel of judges, including Dr. Iona Heath, the President of the College, oversaw the motion "This House would waive patents for anti-retroviral therapies in the developing world." The motion was proposed by Cambridge and opposed by Oxford.

There are estimated to be 29.5 million people infected by Human Immunodeficiency Virus (HIV) in low- and middle-income countries with only 5.3 million of those being treated [2,3]. The effective and equitable distribution of anti-retroviral therapy (ART) to combat Acquired Immune Deficiency Syndrome (AIDS) is consequently of great importance. Furthermore, agonies over resource distribution have been aggravated by the current global economic climate, which has resulted in a reduction in aid available for the provision of anti-retroviral therapy. While not representing a cure, treatment with ARTs means that AIDS becomes a chronic disease, extending life by decades. The United Nations were unequivocal in the urgent imperative that this problem be tackled, stating in Millennium Development Goal 6B that we must "achieve, by 2010, universal access to treatment for HIV/AIDS for all those who need it" [4]. Tragically, the rate of new infections continues to vastly outstrip the provision of treatment, and the countries facing the heaviest burden of disease are also those with the fewest resources available to tackle the problem. The debate questioned whether a patent waiver would address this shortfall of healthcare provision in developing countries.

This report will summarise the arguments of Cambridge University's team in favour of waiving patents for anti-retroviral therapies and the differing analysis of Oxford University's team suggesting that this is not the first necessary step.

## Proposition

Cambridge University proposed that all patents outside those held by the UNITAID patent pool be waived in the least developed countries. Pharmaceutical companies could avoid the waiver and gain revenue from their products by submitting patents to the patent pool. Patent pools have been promoted by several global health organisations, including the World Health Organisation (WHO) and Médicins Sans Frontières [5] as a means of improving access to medicine in the developing world. A patent pool allows any company to make use of the patents in the pool, for a pre-arranged royalty fee. This facilitates rapid and efficient production of generic drugs by many different manufacturers, whilst still allowing the initial patent holders to achieve some income for their intellectual property. The advantages of this model are threefold: firstly, access to patents by multiple manufacturers increases the opportunity for price competition between drugs. This stands in stark contrast to the current situation of a near-monopoly over all but the oldest ARTs held by just a few pharmaceutical giants. Secondly, a pool of patents facilitates collaborative research efforts into new developments such as combination therapies. Thirdly, this allows the development of local manufacture of ARTs, thereby introducing both the economic and logistical benefits of local drug production.

The single greatest barrier to achieving universal access to ARTs is cost: a monthly dose of maraviroc, an HIV entry inhibitor that came onto the market in 2007, costs approximately $870 [6]. However, the cost of generic ARTs is much lower, due to price competition between manufacturers, and the lack of pressure to recoup losses made during development of the drug. For example, a recent study found that the cost of providing a year's worth of generic ART in Haiti was $1000 [7] -only slightly more than only a month's dose of a single patented drug. Reduced cost is not the only benefit of patent pooling. The procurement of drugs is only the first step in achieving long-term treatment for HIV sufferers; other obstacles to success include logistical difficulties such as heat degradation of many of the patented products and the complexity of multi-drug therapies. Historically, generic products have been manufactured that overcome these problems - in particular new combination therapies that increase patient compliance by simplifying complicated dose regimens and the development of heat-stable therapies. Traditionally, ART drugs needed refrigerated storage, often leading to needless wastage when this storage was either unavailable or unreliable [8]. It is impossible for generic drug companies to apply these developments to drugs that are under patent, and the patent holders do not always consider it sufficiently profitable to do so. Hence if all ARTs are either unpatented or held within the UNITAID patent pool, standardized combination therapies could be made available to the acutely vulnerable groups that are currently not targeted by pharmaceutical companies: pregnant and breast-feeding women at risk of vertically transferring HIV to their infants, children for whom standard therapies are too toxic, and the millions without access to refrigerated drug storage.

Another advantage of patents being waived specifically in developing countries is the opportunity for growth of local manufacture. While it is true that some developing countries currently lack the technological capacity and infrastructure to independently manufacture ARTs, there have been examples of successes in this area in the past, when developing countries have made use of voluntary or compulsory licensing of drugs to permit local production. In South Africa, for instance, voluntary licensing of ARTs for production by the leading generic drug manufacturer, Aspen, resulted in sustainable local production of ARTs with particularly low prices for the public sector [9]. Although many sub-saharan countries would be unable to develop local production, these countries would still be able to import generic drugs legally and hence this could take place until manufacturing ability could catch up. Successful development of local manufacture in some, and eventually many countries could improve accessibility of therapies and streamline domestic procurement systems, having a significant impact upon public health as well as having the potential to create jobs and improve infrastructure.

Waiving patents for ARTs in the developing world would reduce their cost, promote development of innovative solutions to the unique context of different regions, and facilitate more efficient distribution of treatment to those who most need it. The main arguments of the proposition are summarised in table 1.

## Opposition

The team from Oxford University presented the opposition case that waiving patents was not a solution to the mutually agreed problem of ART distribution in the developing world, and went further to argue that waiving patents could be detrimental to the efforts against HIV/AIDS.

Intellectual property rights exist for several purposes, not least of which is to ensure monetary reward for the owner of the patent. Pharmaceutical companies are often characterized as profiteering, more interested in their own gain than in the benefit of the end-users of their products, patients. This characterization is not only accurate but quite appropriate; every company is beholden to its share-holders, and moreover an unprofitable company will simply cease to be. In the case of the pharmaceutical industry, where development and manufacturing are both necessarily highly costly and highly specialized, reducing profitability is potentially harmful to all. To develop pharmaceutical products is to invest millions into an enormously high-risk venture, relying upon the product to pass scrupulous safety and efficacy testing, carried out at great expense to the developer - estimates of overall cost vary from 500 million to over 2,000 million dollars [10]. What does this mean in the context of ARTs?

Drug development for diseases that predominantly affect the developing world is already a precarious business. We see in the case of orphan drugs [11], incentivizing companies to invest in research and development (R&D) for rare or unprofitable diseases is expensive and costly in diplomatic reserve on the part of governments or organisations involved and is rarely successful. Consistently it is apparent that pharmaceutical companies will only invest into R&D for diseases which are considered profitable. To waive patents for ARTs in the developing world would be to wave a banner saying investing in HIV in the developing world is to pour money down the drain- worse, to pour money into your rivals' pockets. It can be argued that the developing world represents an insignificant fraction of the market for ARTs, and that the profit from patents in the developed world would continue to support research and development. There are two problems with this analysis: firstly, the numbers do not hold up. Sub-Saharan Africa alone has over 22.5 million patients infected with HIV compared to 860,000 in the whole of Western Europe [3] - even with poor access to ART, the developing world represents an incredibly significant fraction of the market. Further, only approximately 5.3 million people out of a possible 14.6 million (36%) eligible patients in low and middle income countries received ART in 2009 [12]. This compares to approximately 50,300 out of a possible 64,600 (78%) eligible patients receiving treatment in the UK in the same year [13]. Clearly, the developing world currently represents the largest *potential *marketplace for ARTs.

This brings us to the second problem: as we have already seen in the guise of heat-resistance, problems exist in many developing countries that do not in the developed world. To chip away at profit in the developing world is to disincentivise those best placed to tackle these problems from attempting to do so. Finally, a patent waiver would send the message that treatments which primarily benefit developing nations can never be profitable - hence undermining efforts to encourage investment in this sector. Waiving patents in these countries could be a devastatingly short-termist approach to the problem, in the long-term harming those it is intended to help. There is direct evidence of the harm that can be wrought by a patent waiver - in 2007 Abbott (the world's 10^th ^biggest pharmaceutical company) were so angered by Thailand's decision to ignore patents that they did not apply for licenses to sell their latest products there, one of which was a new heat-resistant formula, which would have been particularly helpful for the hot South East Asian climate.

In the long-term, then, money certainly seems to be the primary problem. But is it the case, as it has been contended, that the cost of ARTs is the greatest barrier to their effective distribution? And are patents to blame for this? There is evidence to suggest that the answer to both of these questions is no.

The Agreement on Trade Related Aspects of Intellectual Property Rights (TRIPS) agreement between World Trade Organisation (WTO) member countries was the first international agreement on intellectual property law. Among other outcomes, TRIPS stipulated that member states respect patent law. Although all provisions apply equally to all members, developing countries were given a transition period, until 2016, before changes to their domestic laws regarding pharmaceutical patents need to be put in place [9], and many countries made use of the transition period. We thus have a model within which to examine the effects of a system whereby patents are enforced in the developed world but not in the developing world. One study examined the patent-status and accessibility of 15 different ARTs in 53 African countries in 2002 and found that the drugs were patented in very few of those countries (median 3; mode 0)[14]. The accessibility of ARTs did not correlate with patent coverage. This suggests that, while the "Big Pharma" giants offer an appealing scapegoat for a global tragedy, the situation is far more complex than it first appears and indeed waiving patents may not be as effective as proposed. The barriers to ART in the developing world are many, and while drug cost is certainly an enormous issue, some developing countries have shown that gains can be made without the drastic and potentially harmful course suggested by the proposition.

This brings us finally to some of the alternative targets for intervention. One of the many difficulties in successful ART is the risk of resistance to first-line therapy developing. Resistance develops most rapidly in the context of unplanned interruptions of treatment, often a consequence of poorly managed procurement strategies in developing countries [15]. Malawi provides us with an excellent model [16] for coping with this problem: developing a nationwide standardized program of therapy simplifies the process of matching supply to demand and of eventual distribution. Another advantage of this system is that it reduces the need for medically trained staff, who face an overwhelming patient to staff ratio in many developing countries. Lay staff can relieve this burden and provide effective care in regions with limited medical resources.

Improving access to ARTs in the developing world is an indisputably important goal. However, to target patents is to miss many of the true barriers to ART access and, in the long-term, may be seriously detrimental. While less intuitively attractive, the application of many smaller, fine-tuned changes such as those to national HIV/AIDS strategies has been a more effective and practical alternative.

## Key issues

The debate centred around three major points of disagreement:

1. What is the true barrier to ART access in the developing world?

2. Would waiving patents increase access?

3. In the long-term, is waiving patents beneficial?

### 1. What is the true barrier to ART access in the developing world?

Poverty is undeniably a barrier to ART distribution in the developing world; limited international and domestic funds available for HIV/AIDS treatment mean that the high prices of many drugs make them quite simply unaffordable. Even the cost of generic drugs, however, often exceeds the means of the millions who subsist on less than $1 a day. The problems are thus multifactorial: as well as financial resources, human resources are strained in countries such as Tanzania or Malawi where the ratio of patients:doctors exceeds 50,000:1 (compared to 440:1 in the UK)[17]. Efficient and specialized management is needed to assess regional needs and to deliver treatments appropriately and sustainably.

### 2. Would waiving patents increase access?

The evidence on this issue appears to be mixed. On the one hand, it is clear that the amount of money available for ARTs in the developing world is limited by national resources and international aid. For this reason, reducing drug costs by waiving patents would be of obvious benefit. This analysis is at odds with evidence available from countries that did not uphold patents prior to the TRIPS agreement [9]. It seems likely, therefore, that waiving patents is not a sufficient intervention to improve access when implemented alone and can even be viewed as having unintentional detrimental consequences [18]. Organisations such as UNITAID support multilateral developments, and measures such as voluntary licensing agreements with which pharmaceutical companies reap the benefits of waiving patents with fewer of the harms outlined by the opposition. It is a combination of measures, from improving infrastructure, education and prevention alongside any patent waivers via voluntary licensing agreements that will ultimately bring most benefit.

### 3. In the long term, is waiving patents beneficial?

The major argument opposing the waiving of patents is the long-term harm to investments in research and development. The costs of developing any drug are enormous, and the risk of the investment is very high. It can be argued that the pharmaceutical industry is already beginning to limit the scope of research and development, by more often investigating permutations of currently successful drugs rather than investigating entirely novel classes. However, the model proposed by Cambridge would not entirely deprive companies of the opportunity for profit- royalties would be paid for any patent submitted to the patent pool. This may therefore ameliorate the harms of profit reduction. Nonetheless, this remains a significant consideration in the approach to HIV/AIDS in the developing world. It may be that solutions engineered through co-operation with the pharmaceutical industry are a good compromise. By running an index of pharmaceutical companies and highlighting their good and poor practices, The Access to Medicine Foundation encourages socially responsible behaviour from Big Pharma - for example the issuing of five voluntary licensing agreements to generic drug companies in sub-Saharan Africa by Merck&Co [19]. Pharmaceutical companies are also not the only route for therapeutic innovation, and the work of groups such as the Bill and Melinda Gates Foundation in seeking a vaccine against HIV [20] provides another avenue for future progress. This approach, as well as other projects such as public-private partnerships could be a very rewarding strategy and merit more attention.

## Discussion

After lengthy deliberation, the judges narrowly awarded victory to Oxford. The judges praised the knowledge and charisma of all the debaters and praised the Cambridge team for their model. The Oxford debaters were able to show successfully in this debate that waiving patents with the method favoured by the proposition might have three key unintended consequences. Firstly, there might be a reduction in R&D in future or more innovative ARTs. Secondly, the move might send a message that any drugs for developing countries will never be profitable. Thirdly, twisting the arm of pharmaceutical companies might make them resentful and less amenable to future public-private initiatives. Furthermore, they were able to explain that there are a number of other barriers to achieving distribution of ARTs. These included additional to cost of drugs, distribution logistics, complex therapeutic regimes, heat degradation of medicines, poor political will and lack of healthcare infrastructure and patient education.

The issues brought up at the debate are applicable to many other infectious diseases in the developing world and there are a number of key points to consider. The ability to purchase ARTs, or indeed any other therapy, in a sustainable manner does require substantial revenue to be raised; however, problems of access do not depend exclusively on having the money available to pay for drugs. In reality, obstacles to progress are often multifactorial. Professor Thomas Pogge has proposed a model similar to that described by the Proposition. He suggests the creation of a Health Impact Fund, where companies that signed up would forgo monopoly pricing, offering their product at cost price, in exchange for a reward based on the global health impact of the new medicine. He highlights the efficiency of this mechanism in maximising spending on impactful solutions, and suggests that the wealthy will also benefit through fewer drug resistant disease strains and potentially through lower costs [21].

Research in areas that may not be profitable in developed countries needs to be encouraged. Encouraging private sector involvement is one way to encourage innovation; however, just because research is being produced by means of profit-making institutions does not imply that this is the only (or best) way to encourage the development of new treatments. The Opposition did not provide any evidence for a causal relationship between the profit motive and new drugs.

While it may be a laudable aim to encourage growth within the economies of developing countries, Kenneth Shadlen suggests that it may be difficult for countries to develop their medical industries around TRIPS. Incentives are misaligned by new regulation, such that those firms who have capacity to produce do not want to, since antiretrovirals carry a high cost burden and will be sold at low prices. Generics firms with capacity are more likely to enter the specialised generics market, where resources must be invested to produce the drug but where the capacity for profit is high. Conversely, those firms who would like to enter the antiretroviral generic market typically will not have the capacity to do so. These barriers to entry are in fact part of the rationale for TRIPS, in providing the research-based pharmaceutical sector with protection from generic firms [22].

The problems of infrastructure include transport and storage. Building healthcare systems in developing countries requires sustained political will. Non-governmental organisations such as The Bill and Melinda Gates Foundation are vital for innovation, for the greatest risks have the greatest potential for reward.

At the heart of this debate lies a tension between intellectual property rights and human rights. There is always a balance to strike between ensuring there are rewards for innovation and ensuring that people suffering from diseases have access to treatments that facilitate better health and enjoyment of other human rights. There is no easy answer to this question; however, the substance of this debate provokes many questions as to the ethical and equitable distribution of resources for the benefit of the world's population.

## About the Debate

The Varsity Medical Debate was started in 2008 with the aim of creating a discourse on pertinent ethical issues among medical students. Utilising the age-old rivalry between the two Universities, it encourages medics from both Oxford and Cambridge to consider and articulate the crucial arguments surrounding questions that will feature heavily in their future careers. Whilst the debaters may not necessarily agree with their allocated side, the debate format forces them to articulate a certain school of thought and present the key arguments to support this.

As well as being judges on the logic of the analysis provided and evidence presented, participants are judged on style and delivery. As such, although Oxford were awarded a victory for opposing the motion, this does not mean that they or the judges were making a truth claim regarding the problem of access to ARTs.

This meeting report aims to chronicle the proceedings of the debate and explain both sides of the argument to allow for consideration of this important issue.

## Competing interests

The authors declare that they have no competing interests.

## Authors' contributions

FC wrote the first draft of the manuscript. FC, RT and SB revised and edited subsequent drafts. All authors read and approved the final manuscript.
